# Anthocyanin-Rich Purple Plant Foods: Bioavailability, Antioxidant Mechanisms, and Functional Roles in Redox Regulation and Exercise Recovery

**DOI:** 10.3390/nu17152453

**Published:** 2025-07-28

**Authors:** Jarosław Nuszkiewicz, Joanna Wróblewska, Marcin Wróblewski, Alina Woźniak

**Affiliations:** Department of Medical Biology and Biochemistry, Faculty of Medicine, Ludwik Rydygier Collegium Medicum in Bydgoszcz, Nicolaus Copernicus University in Toruń, 24 Karłowicza St., 85-092 Bydgoszcz, Poland; joanna.wroblewska@cm.umk.pl (J.W.); marcin.wroblewski@cm.umk.pl (M.W.); al1103@cm.umk.pl (A.W.)

**Keywords:** bioavailability, dietary polyphenols, exercise recovery, functional foods, oxidative stress, redox homeostasis

## Abstract

Anthocyanin-rich purple fruits and vegetables—such as blackcurrants, blueberries, purple sweet potatoes, and red cabbage—are increasingly recognized for their health-promoting properties. These natural pigments exert antioxidant and anti-inflammatory effects, making them relevant to both chronic disease prevention and exercise recovery. This review critically examines current evidence on the redox-modulating mechanisms of anthocyanins, including their interactions with key signaling pathways such as Nrf2 and NF-κB, and their effects on oxidative stress, mitochondrial function, vascular homeostasis, and post-exercise adaptation. Particular attention is given to their bioavailability and the challenges associated with their chemical stability, metabolism, and food matrix interactions. In light of these factors, dietary strategies and technological innovations to improve anthocyanin absorption are also discussed. The synthesis of preclinical and clinical findings supports the potential of anthocyanin-rich foods as functional components in health optimization, athletic performance, and recovery strategies.

## 1. Introduction

**Figure 1:**
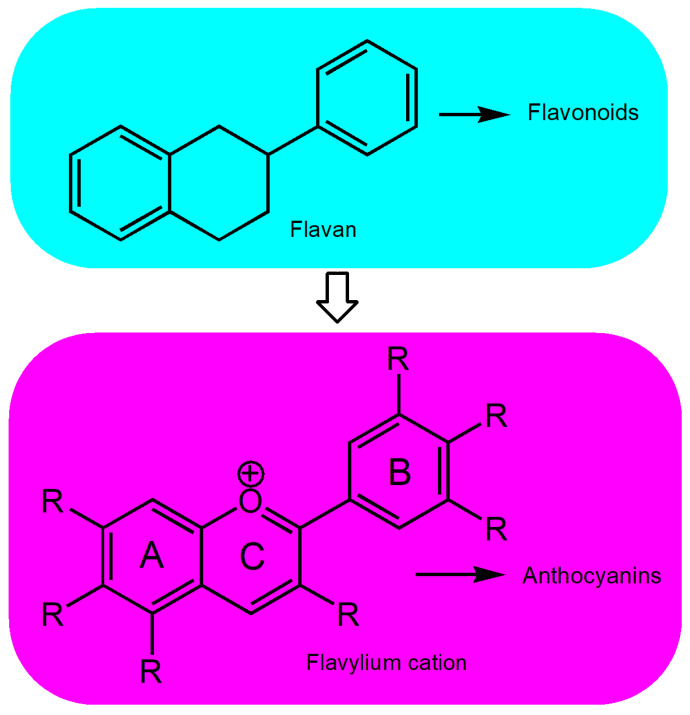
Flavan as the structural unit of flavonoids, and the flavylium cation as the structural unit of anthocyanins. R represents possible substituents such as –H, –OH, or –OCH_3_.

Flavonoids, also known as flavones, are a diverse group of natural compounds found in plants. These polyphenolic compounds serve various biological functions in both plant and animal systems. Well-known flavonoids include catechins, quercetin, rutin, isoflavones, anthocyanins, and hesperidin [1]. Structurally, these compounds are benzopyran derivatives characterized by the 2-phenyl-3,4-dihydro-2H-chromene skeleton. Anthocyanins, a subgroup of flavonoids, consist of a sugar moiety and an aglycone responsible for their coloration, termed anthocyanidins. The aglycone is a polyhydroxy and polymethoxy flavylium cation comprising an aromatic A-ring linked to an oxygen-containing heterocyclic C-ring. This C-ring, in turn, connects via a carbon–carbon bond to another aromatic ring designated as B (Figure 1). In plants, this aglycone exists in mono-, di-, or tri-glycoside forms [2].

Anthocyanins are primarily known for providing color to plants. The pigmentation of fruits and vegetables often indicates their antioxidant levels, with vibrant colors like those in black grapes, blueberries, and red cabbage being particularly beneficial due to their high anthocyanin and phenol content [3]. For example, the purple sweet potato is recognized for its exceptional antioxidant activity, primarily attributed to its high anthocyanin content (peonidin-3-caffeoyl-p-hydroxybenzoylsophoroside-5-glucoside) [4]. However, caution is needed, as some reports overestimate anthocyanin content based solely on color. This is evident in many heirloom tomato varieties labeled as ‘purple’ or ‘black,’ which actually lack anthocyanins [5]. Additionally, some purple-hued fruits and vegetables, such as plums, eggplants, and red onions, have lower antioxidant effects, likely because anthocyanins are mainly in their skins, with the inner flesh differing significantly in content and color [3]. The beneficial properties of anthocyanins, including their therapeutic effects, are largely due to their antioxidant and anti-inflammatory actions [6]. The antioxidant activity of anthocyanins depends on their chemical structure, resulting from their high reactivity as hydrogen or electron donors and their ability to chelate transition metal ions via hydroxyl groups [7].

Oxidative stress and chronic low-grade inflammation are widely recognized as fundamental contributors to the pathophysiology of numerous chronic diseases, including obesity, metabolic syndrome, type 2 diabetes, and cardiovascular disorders [8]. Disruption of redox homeostasis triggers the activation of redox-sensitive signaling cascades, such as nuclear factor kappa B (NF-κB), c-Jun N-terminal kinase (JNK), and p38 mitogen-activated protein kinase (p38 MAPK), which amplify inflammatory responses, impair mitochondrial function, and alter metabolic regulation [9,10]. These processes form a vicious cycle that accelerates tissue damage and disease progression. As a result, they have become key targets for preventive dietary strategies aimed at restoring redox balance and modulating inflammation [11,12]. In this context, bioactive plant compounds with antioxidant and anti-inflammatory properties, such as anthocyanins, have received growing attention [13]. Their ability to influence molecular pathways involved in oxidative stress and inflammation suggests a potential role in supporting metabolic health, reducing disease risk and enhancing physiological resilience.

As dietary antioxidants, anthocyanins may help prevent diseases like cancer, diabetes, inflammation, and various neurological and cardiovascular conditions. Foods and supplements rich in anthocyanins enhance recovery after exercise, increasing their popularity in sports nutrition. Anthocyanin supplementation may reduce post-exercise muscle soreness and improve performance parameters [7]. Although anthocyanins are not classified as essential nutrients, such as vitamins or minerals, their bioactive properties, particularly antioxidant and anti-inflammatory effects, have been associated with a range of potential health benefits. The potential health benefits of anthocyanins make them a promising area of research. Future studies should aim to identify the optimal dosage and consumption patterns for maximizing their health benefits. Additionally, research should focus on improving the bioavailability and stability of anthocyanins in food products and supplements. Investigating the long-term effects of anthocyanin consumption and their interactions with other dietary components will provide valuable insights into their role in human health.

The study of anthocyanins dates back to the early 20th century when researchers first identified their role in plant pigmentation. Since then, extensive research has highlighted their antioxidant properties and potential health benefits. In recent years, there has been a growing interest in the role of anthocyanins in sports nutrition and chronic disease prevention. Current trends in research are focusing on understanding the molecular mechanisms behind their health benefits, improving their bioavailability, and exploring their therapeutic potential in various medical conditions.

This narrative review aims to explore and critically evaluate the ergogenic and health-promoting effects of consuming anthocyanin-rich purple vegetables and fruits. Particular attention is given to their ability to modulate redox homeostasis, reduce exercise-induced oxidative stress and inflammation, and support recovery and physiological adaptation through antioxidant and signaling-related pathways. Anthocyanins are increasingly investigated not only for their radical-scavenging capacity, but also for their involvement in vascular function, mitochondrial dynamics, and inflammatory regulation-all of which are highly relevant to sports nutrition and functional food research. A comprehensive literature search was conducted using PubMed, Scopus, Web of Science, and Google Scholar to identify English-language articles published between 2000 and 2025. Search terms included “anthocyanins,” “oxidative stress,” “inflammation,” “exercise recovery,” “bioavailability,” “sports nutrition,” “purple vegetables,” and “ergogenic aids”. Eligible studies included original research, meta-analyses, and reviews investigating the effects of anthocyanin-rich foods on oxidative stress, inflammation, exercise performance, recovery, or anthocyanin bioavailability in human or animal models. Studies were excluded if they focused solely on non-anthocyanin flavonoids, lacked peer review, or did not include sufficient methodological detail.

## 2. Oxidant–Antioxidant Balance and Mechanisms of Redox Regulation

Energy needed by humans for all forms of activity, including sports, is released at the cellular level, in the process of respiration. Most organisms, humans included, execute aerobic respiration, during which the respiratory substrate is oxidized in the presence of oxygen. Although oxygen is essential for aerobic metabolism, its role is not unequivocally positive [14]. Reactive oxygen species and reactive nitrogen species (RONS), which include free radicals, are formed as by-products of aerobic metabolism in the body. Free radicals are atoms or molecules capable of independent existence (hence the name “free”) that have one or more unpaired electrons in the valence shell or outer orbit (hence “radical”) [14,15]. The term “reactive forms”, on the other hand, is a broader concept that includes not only free radicals, but also non-radical unstable derivatives, also referred to as oxidants [16]. What results from the presence of an unpaired electron is high reactivity of free radicals, which is higher than in non-radical species; they are also less stable [16]. To regain stability they can abstract electrons from other compounds. A molecule from which an electron is abstracted becomes a free radical [17]. Oxygen and nitrogen derivatives that are free radicals include the following: superoxide (O_2_^•−^), hydroxyl radical (OH^•^), peroxyl radical (ROO^•^), and nitric oxide (NO^•^). The non-radical species include the following: hydrogen peroxide (H_2_O_2_), singlet oxygen (^1^O_2_), ozone (O_3_), nitrous acid (HNO_2_), and peroxynitrite (ONOOH) [18]. In addition to the respiration process, RONS are also produced during other biological processes occurring in the human body, among others, digesting food, metabolizing therapeutic agents, and converting fats into energy [19]. RONS are therefore produced continuously by cells. Their main sources in the cell are the following: mitochondria (electron transport chain), cytoplasm (reactions catalyzed by NO synthase and xanthine oxidase), endoplasmic reticulum (reactions involving cytochrome P450, CYP), peroxisomes, and plasma membrane (reactions involving NADPH oxidases, NOXs) [20]. Their proper generation in cells and maintenance at low levels is essential for a number of processes, such as activation of several transcriptional factors, protein phosphorylation, immunity, differentiation, and apoptosis [21]. RONS formed in the cell in excess can react with fundamental biomolecules (proteins, lipids, DNA) leading to irreversible damage to cellular structures, resulting in cell death and organ failure [16,22]. Lipids are particularly sensitive to oxidants. Hydroxyl radical or peroxynitrite, for example, can induce lipid peroxidation (oxidation of polyunsaturated fatty acids or other lipids), which leads to damage of cell membranes and lipoproteins [21]. The final, so-called secondary products of the peroxidation process include hydroxynonenal (4-HNE), malondialdehyde (MDA), and isoprostanes [23]. The aforementioned compounds are the main markers used to assess the intensity of the lipid peroxidation process [24].

Cells are protected against the harmful effect of RONS thanks to the activity of antioxidant enzymes. The most important enzymes involved in their neutralization include superoxide dismutase (SOD), glutathione peroxidase (GPX), and catalase (CAT). Three isoforms of SOD have been identified in mammalian cells, namely, mitochondrial, cytosolic, and nuclear Cu/Zn-SOD (SOD1), mitochondrial Mn-SOD (SOD2), and extracellular SOD (ec-SOD or SOD3) [14]. These enzymes catalyze the conversion of superoxide radicals to oxygen and hydrogen peroxide in the dismutation reaction. Glutathione peroxidase is a family of selenoenzymes that catalyze the reduction of H_2_O_2_ or organic hydroperoxides to water or corresponding alcohols. Eight types of GPX (GPX1–GPX8) have been identified in mammals; GPX6 is only expressed in humans [25]. One of the most important antioxidant enzymes is CAT. This enzyme, in a two-step reaction, breaks down two hydrogen peroxide molecules to an oxygen molecule and two water molecules. Considering the differences in sequence and structure of CATs, three different types of enzyme have been distinguished: monofunctional heme-containing enzyme, heme group-containing bifunctional catalase-peroxidase, and Mn-containing CAT. Monofunctional heme-containing CAT occurs in humans [26]. Non-enzymatic scavengers are also involved in maintaining redox homeostasis. Only a few of them are produced in the human body, e.g., glutathione and uric acid [18]. For the most part, they are supplied with food, and their main source is fruit and vegetables. Among the most important natural antioxidants are carotenoids (carotenes and xanthophylls), phenolic compounds (anthocyanins, flavonoids, stilbenes, and lignans), and vitamins (vitamin E and C) [18,27,28].

Maintaining the balance between the RONS that are formed and the antioxidants that participate in their removal is an essential condition for health maintenance [18]. Physiological, low or mild level of RONS and their contribution to biochemical processes is referred to as oxidative eustress or “good stress”. An oxidant–antioxidant imbalance involving excessive production of RONS and/or impaired functioning of antioxidant mechanisms is referred to as oxidative stress (“bad stress”) [20,29].

The oxidant–antioxidant balance is affected by various factors and processes, including environmental stressors, nutrition, pathogenic microbial infections, and a wide variety of diseases [30]. Maintaining redox homeostasis is particularly critical in the context of exercise, where increased metabolic activity leads to elevated RONS production. Dietary antioxidants, such as anthocyanins, may play a compensatory role in supporting this balance and preventing oxidative damage.

## 3. Exercise-Induced ROS Production and Physiological Adaptation

The main source of RONS during physical exercise appears to be the skeletal muscle, although increased production of these toxic oxygen and nitrogen derivatives has also been shown in the endothelial cells associated with contracting muscles [31]. RONS generation occurs in both contracting and relaxing skeletal muscles [32]. Initially, it was thought that the production of RONS during physical exercise in skeletal muscles was mainly responsible for the mitochondrial electron transport chain. However, scientific research conducted in recent years shows that mitochondria during active State 3 respiration generate less O_2_^•−^ compared to basal State 4 respiration; therefore, they do not appear to be a major source of oxidants during exercise [33]. An important role in the generation of RONS in muscle fibers during physical exercise appears to be played by phospholipase A_2_ and NADPH oxidases (NOX2 and NOX4) present in sarcolemma, mitochondria, T-tubules, and sarcoplasmic reticulum [33]. Phagocytic cells may also be an important source of RONS during physical exercise, namely, neutrophils and macrophages, which are activated during muscle injuries [34]. In a process referred to as oxidative burst, these cells, with the participation of membrane-localized NADPH-oxidase (NOX2), generate O_2_^•−^, which undergoes dismutation to H_2_O_2_. These oxidants can then participate in the production of other RONS, leading to tissue damage, or driving other immune cells to apoptosis [35].

The relationship between oxidant–antioxidant balance and physical exercise is extremely complex and depends on gender, age, training level of the body, and the intensity and duration of exercise [36]. After acute exercise, for example, an increase in the concentration of MDA in the plasma of eight healthy male soccer players has been observed [37], as well as an increase in the levels of 8-isoprostanes, SOD, and CAT in the plasma/blood serum of eight healthy men [38]. Higher plasma MDA concentrations were also found in 20 healthy, untrained men after treadmill running. Increased MDA concentrations were observed after both moderate-intensity (of 65% maximal oxygen uptake) and high-intensity (85% maximal oxygen uptake) running. However, men in the “high-intensity” group had higher post-exercise MDA concentrations than in the plasma of men in the “moderate-intensity” group. A similar nature of changes was also observed in the plasma SOD activity of these men [39]. However, improvement in the functioning of antioxidant mechanisms, leading to a reduction in the intensity of lipid peroxidation, was demonstrated in long and middle-distance athletes during a sport season [40]. After 10 weeks of resistance training, also in older adults, a decrease in MDA concentration was observed and a concomitant increase in serum GPX activity and levels of total antioxidant capacity (TAC) [41]. In contrast, Sielski et al. [42] showed no statistically significant changes in the concentration of the thiobarbituric acid reactive substances—TBARS (expressed in nmol MDA)—in blood plasma of 27 female volleyball players from an amateur sports club after a single exercise test (30  min run) on an antigravity treadmill (AlterG) or on a classic treadmill (distance of 5  km at an average speed of 10  km/h). TBARS concentration in erythrocytes in turn increased 30 min after the running test on the classic treadmill. A total of 24 h after the end of exercise on the classic treadmill, SOD activity in erythrocytes increased, while GPX and CAT activity did not change in a statistically significant manner. Although results vary by training status and protocol, most studies confirm a rise in oxidative markers following acute high-intensity exercise, highlighting the role of RONS as mediators of physiological stress.

The beneficial effect of physical activity on human health is well known. However, the molecular mechanisms explaining the effect of exercise on the human body are very complex and have not been fully understood. Physical exertion leads to many adaptive changes, bringing multifaceted health benefits. Regular moderate-intensity exercise is widely accepted as a preventive and therapeutic measure for, among other things, cardiovascular diseases, metabolic diseases, pulmonary diseases, or neurological diseases [43]. However, high-intensity physical exertion, especially by untrained individuals, can lead to adverse cardiovascular events, including myocardial infarction [43]. The research results presented by scholars on redox balance after physical exertion/exercise are quite diverse and sometimes even contradictory. However, a certain regularity can be observed. Moderate and regular physical activity seems to be beneficial for maintaining redox balance, where strenuous and acute bouts of anaerobic and aerobic exercise in turn induce oxidative stress. On the other hand, however, increased generation of RONS is necessary to induce adaptation mechanisms [36]. For example, it has been shown that in skeletal muscle and vascular endothelium, under the influence of physical exercise, the expression of nuclear erythroid-2 like factor-2 (Nrf2) increases. This transcription factor induces antioxidant gene transcription, thereby promoting the protection of skeletal muscle from RONS-induced damage [31]. The changes occurring at the level of cells and organs during exercise, including the generation of RONS, are attempted to be explained, among other things, by the induction of hormetic mechanisms [44]. According to the concept of hormesis, exposure to a low dose of a harmful agent (as opposed to a high dose of that agent) can have a beneficial effect on the long-term well-being of the body. RONS function as regulatory mediators in signaling processes; therefore, their concentration must be maintained within a certain range. Excessively low levels of RONS can lead to the lack of the benefits of hormesis [36]. Studies confirm that RONS may be potentially related to the beneficial effect of exercise on the human body [45]. This regulatory role of RONS is not yet fully understood. Much interest is now being shown in studies looking for safe substances that can promote exercise capacity and/or reduce exercise-induced damage. They are particularly valuable for achieving better athletic performance or increasing the possibility of using exercise in the treatment of certain diseases. It is possible that substances that affect RONS levels in cells should be the target of this type of research. These findings support the relevance of dietary antioxidant strategies, including anthocyanin-rich foods, to support redox regulation in active individuals.

## 4. Sources, Composition, and Structure of Anthocyanins in Purple Plant Foods

### 4.1. Chemical Structure and Biosynthesis of Anthocyanins

**Figure 2:**
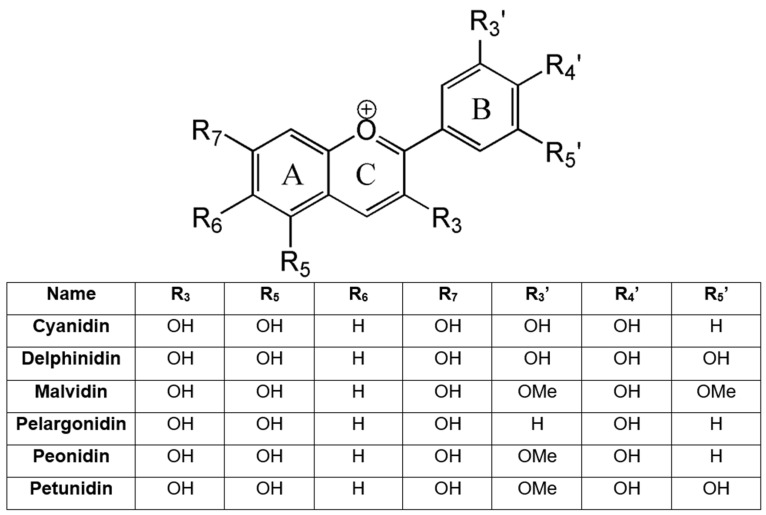
Differences in the chemical structure of anthocyanidins occur most commonly among plants [2].

Anthocyanins are the primary representatives of flavonoids found in purple vegetables and fruits. This group of antioxidant flavonoids are secondary metabolites occurring in higher plants, imparting colors to flowers and fruits. The most common in higher plants in common aglycone type are peonidin, delphinidin, cyanidin, malvidin, pelargonidin, and petunidin (Figure 2) [46].

The color differences in the same plant species mainly stem from the content and structure of the anthocyanin [47]. The MYB-bHLH-WD40 (MBW) protein complex plays vital role in regulating the anthocyanin biosynthesis pathway in plants [48,49]. Anthocyanin biosynthesis is a complex process, which is regulated by multiple genes, including structural and regulatory genes [50,51]. The structural genes directly encode a number of enzymes, including chalcone synthase (CHS), chalcone isomerase (CHI), flavonoid 3-hydroxylase (F3H), flavonoid 3′-hydroxylase (F3′H), flavonoid 3′5′-hydroxylase (F3′5′H), phenylalanine ammonia lyase (PAL), dihydroflavonol reductase (DFR), anthocyanidin synthase (ANS), and anthocyanin 3-O-glucosyltransferase (UFGT), and they can promote or inhibit the synthesis of anthocyanins [47,50,51,52]. F3′H specifically targets the B-ring of dihydrokaempferol for hydroxylation at the 3′ position, leading to the formation of dihydroquercetin. Meanwhile, F3′5′H performs hydroxylation at both the 3′ and 5′ positions on the B-ring, resulting in the production of dihydromyricetin. The hydroxylation patterns, which are determined by F3′H and F3′5′H, direct the specific biosynthesis of anthocyanins [52]. F3′H predominantly catalyzes the formation of cyanidin-based anthocyanins, whereas F3′5′H is crucial for the synthesis of delphinidin-based anthocyanins. In the context of plant coloration, the ratio of F3′5′H to F3′H is a critical factor; plants exhibit more intense purple colors when the F3′5′H/F3′H ratio is higher [52,53]. The regulatory genes play an important role in participating in the formation of the plant color at the transcriptional level by regulating the expression of structural genes [49,50]. The gene expression involved in the regulation of anthocyanin biosynthesis is associated with genotype of plant [5,51,52,54,55,56].

### 4.2. Botanical Sources of Purple Anthocyanins

In recent years, the study of bioactive compounds derived from purple vegetables and fruits has increased. Accumulation anthocyanins are known to be involved in the purple pigmentation in plants. Popular purple vegetables cultivated in Europe include purple broccoli (*Brassica oleracea* var. *italica*), purple Brussels sprouts (*B. oleracea* var. *gemmifera*), red cabbage with purplish leaves (*B. oleracea* var. *capitata*), purple cauliflower (*B. oleracea* var. *botrytis*), purple-leaf kale (*B. oleracea* var. *acephala*), purple kohlrabi (*B. oleracea* var. *gongylodes*), onions (*Allium cepa* L.), and purple beans (*Phaseolus vulgaris* L.) [57]. Among Solanaceae crops, aubergine (*Solanum melongena* L.) is the only species indigenous to the Old World, specifically Southeast Asia. Other purple cultivars from this family—such as purple potatoes (*S. tuberosum* L.), tomatoes (*S. lycopersicum* L.), and capsicums (*Capsicum annuum* L.)—are also grown, but their cultivation in Europe is limited. These varieties are more commonly exported from South America [57]. Purple varieties of carrots (*Daucus carota* L.) belong to the family *Apiaceae*, and are widely cultivated in Turkey, India, Afghanistan, Egypt, and India [58]. Purple fruits commonly grown in Europe include European plum (*Prunus domestica* L.), purple raspberries (*Rubus idaeus* L.), purple aronia berries (*Aronia prunifolia* L.), bilberries (*Vaccinium myrtillus* L.), and blackberries (*Rubus fruticosus* L.*)*. Highbush blueberries (*Vaccinium corymbosum* L.) originate from North America [59]. Purple grapes (*Vitis vinifera* L.) can thrive in a wide range of climatic conditions and successfully grow in both the low temperatures of northern regions and parts of the tropics. [60]. Purple–black fruits of elderberries (*Sambucus nigra* L.) are considered one of the richest sources of anthocyanins and are cultivated in western and central Europe as well as in North Africa, Scandinavia, and Great Britain [61].

In the plant kingdom, pigments are crucial components that manifest vividly in various parts, such as fruits, seeds, leaves, and flowers. Among these pigments, anthocyanins play a prominent role in producing a wide spectrum of colors that can vary from delicate pinks to deep blacks. The variety of colors results from the degree of oxygenation of the anthocyanidin and the number of substituents present in its chromophores [62]. Cyanidin and delphinidin are most common anthocyanins and impart plants a red to purple or blue–purple color [62,63,64]. Peonidin gives a reddish-purple color, and petunidin and malvidin bluish-purple [63,64]. Pelargonidin, produces orange to red colors but can contribute to purple hues in combination with other pigments [62,63,64]. The key types of anthocyanins found in purple plants, along with examples of the plants, are presented in Table 1. The specific content and composition of anthocyanins can vary depending on factors like plant species and genotype, environmental conditions, and cultivation practices [6,47]. Although the anthocyanin content in plants can change and degrade, the anthocyanin profile will be unique, and its qualitative pattern is characteristic [6].

### 4.3. Stability, pH Effects, and Copigmentation Mechanisms

**Figure 3:**
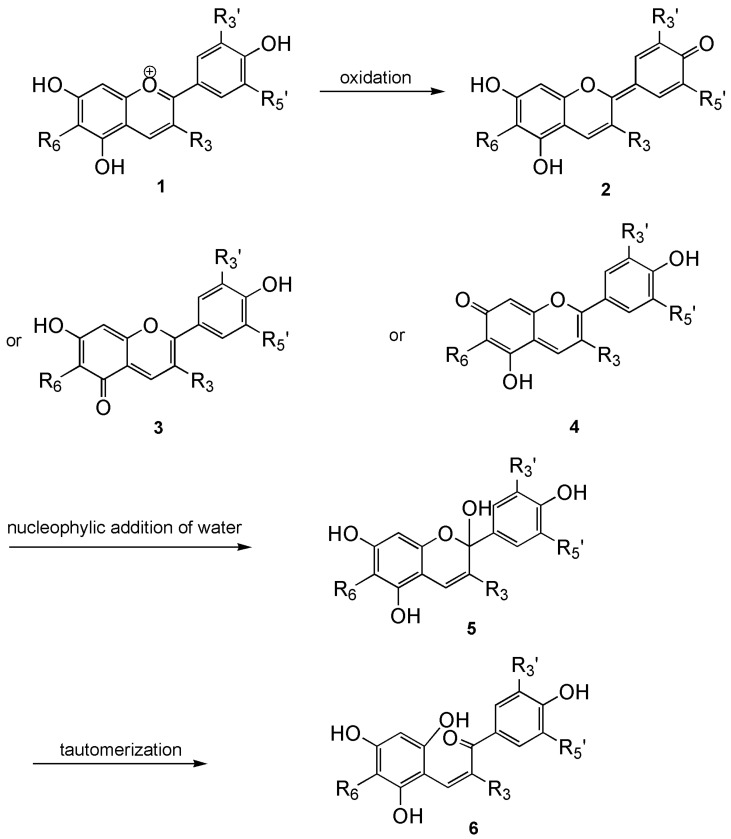
Forms of anthocyanidins depending on pH: **1**—flavylium cation, **2**,**3**,**4**—quinoidal bases, **5**—carbinol pseudo-base, **6**—chalcone.

Consumer demand for plant-derived natural pigments continues to grow. Due to their strong pigmentation and excellent water solubility, anthocyanins are among the most important natural food pigments and are labeled as E163 under the European Coding of Food Additives [81,82]. The stability and color of anthocyanins are influenced by several factors, including pH levels, exposure to light, temperature variations, and their molecular structure. In acidic conditions, anthocyanins exhibit a red hue, which shifts to blue as the pH increases. Anthocyanins that are acylated are known for their enhanced stability and greater antioxidant capabilities compared to their nonacylated counterparts [83]. Anthocyanins demonstrate increased stability in acidic environments due to the formation of the flavylium cation, their most stable form. In slightly acidic or neutral conditions, unstable equilibrium forms of anthocyanins dominate, such as quinoidal bases, carbinol pseudo-base, and chalcone (Figure 3) [83,84]. As the pH increases, the proportion of the red form of the flavylium cation decreases, and in a neutral environment, due to the increased proportion of other equilibrium forms, mainly deprotonated quinoidal bases, the resulting color of the solution is violet. In an alkaline environment, solutions turn blue [64,83]. In the natural environment of anthocyanins, their color depends not only on pH but also on the phenomenon of copigmentation. Copigmentation involves the enhancement or alteration of color by the presence of other pigments belonging to the flavonoid group and metal ions, which can complex with the hydroxyl groups of anthocyanidins contained in anthocyanins [64]. The stability of these compounds depends on modifications of the chemical structure through methylation or acylation of hydroxyl groups, typically in the B-ring, and glycosylation at positions 3 of the C-ring, 5, or 7 of the A-ring. Acylated anthocyanins are known for their increased stability and greater antioxidant capacity compared to their non-acylated counterparts [83]. Glycosylation occurs using monosaccharides (e.g., glucose, arabinose, galactose, xylose, rhamnose), disaccharides (sophorose, rutinose, sambubiose), less commonly trisaccharides [64].

### 4.4. Applications, Intake, and Bioavailability Considerations

Anthocyanins are employed as coloring agents across a wide array of products, including fruit preserves, sugary sweets, dairy items, dry blends (like acid dessert mixes and drink powders), frozen treats (such as ice creams), baked goods, and various beverages. Soft drinks, in particular, are an ideal application for anthocyanins due to their acidic environment (pH below 3.5), which enhances the water solubility of anthocyanins, making them highly effective as natural colorants in these beverages [56]. Purple carrots and purple potato have received much interest as natural sources of anthocyanins for application in the food industry. Anthocyanins from purple carrots and potatoes are more stable than those found in many other purple fruits and vegetables [49,85]. In potato anthocyanins, this is related with highly stable glycosides with sophoroside at the C-3 position and glucoside at the C-5 position of aglycones, along with the presence of organic acids [85]. However, the use of anthocyanins as natural colorants in foods such as cakes and yogurts is constrained due to their limited resistance to processing, formulation, and storage conditions [86]. While anthocyanins are widely recognized for their role in providing color, increasing interest surrounds their potential health benefits as dietary antioxidants and in the prevention of various conditions including cancer, diabetes, and inflammation, as well as neurological and cardiovascular diseases [6,86]. The research indicates that derivatives of cyanidin, delphinidin, and malvidin are the anthocyanins most frequently consumed, representing approximately 45%, 21%, and 15% of total anthocyanin intake, respectively [83]. The abundant delphinidin and cyanidin glycosides, due to the presence of three and two hydroxyl groups in the B ring, respectively, are usually less stable compared to anthocyanins with only one hydroxyl group in the B-ring, such as malvidin and peonidin. The increased polarity suggests a higher reactivity in the presence of reactive oxygen species, making polar anthocyanins potentially more rapidly consumed in oxidative environments [86].

The levels of anthocyanin consumption differ across various countries and regions, influenced by dietary habits and gender differences. In Australia, the average daily anthocyanin intake is around 24.2 mg. In Europe, the intake ranges from 19.8 to 64.9 mg for men and 18.4 to 44.1 mg for women [83]. Among flavonoids, anthocyanins have one of the lowest bioavailability rates, estimated at less than 1–2% [87]. The challenges to anthocyanin bioavailability are attributed to their instability amidst pH fluctuations, as well as degradation by microbes and enzymes during their passage through the gastrointestinal system [87]. A substantial 60% to 90% of anthocyanins can be lost from the upper gastrointestinal tract in as little as four hours after consumption [87]. In the oral cavity, the presence of salivary amylases at a neutral pH may initiate early degradation of anthocyanins. In the acidic environment of the stomach, anthocyanins assume the positively charged flavylium form, facilitating rapid absorption (around 25%). Interactions between anthocyanins and gastric enzymes like pepsin, lipase, and amylase could lead to the formation of stable complexes, which influences their metabolism and ultimately reduces their bioavailability [87]. In the small intestine, where pH levels approximate 7, a range of structural forms of anthocyanins-flavylium, quinoidal bases, hemiketal, and chalcone, can be found, with the quinoidal and/or hemiketal structures often being more prevalent. The colon, which harbors a vast and diverse microbiota with concentrations of 10^12^ to 10^14^ CFU/mL plays a crucial role in the extensive catabolism of anthocyanins, which is why anthocyanins undergo further degradation within the colon’s microbial environment. This degradation process leads to the formation of various phenolic acids. Anthocyanins’ low bio-accessibility and bioavailability significantly limits their potential in disease prevention and treatment [87]. Therefore, the main challenge in using anthocyanins as natural dyes in the food industry is their low stability.

## 5. The Effects of Anthocyanin-Rich Foods on Oxidative Stress and Exercise Recovery

Studies have shown that purple sweet potatoes are effective in reducing oxidative stress and accelerating post-exercise recovery. Dewangga et al. [88] highlighted that purple sweet potatoes with an anthocyanin content of 286.6 mg per 100 g could prevent oxidative stress in male Wistar rats after high-intensity exercise. A daily dose of 3.6 mg of purple sweets reduced the level of MDA (malondialdehyde) and significantly increased the activity of SOD (superoxide dismutase). Chang et al. [89] conducted a study on the impact of a seven-day diet consisting of purple sweet potato leaves on oxidative stress induced by running exercises in a group of untrained, young men. The consumption of purple sweet potato leaves notably raised the levels of total polyphenols and significantly reduced the concentrations of plasma protein carbonyl and TBARS. In another human trial, Chen et al. [90] demonstrated that consuming 200 g per day of purple sweet potato leaves, with a polyphenol content of 902 mg, for two weeks, increased the total phenol levels in plasma and improved LDL’s resistance to oxidation, while also reducing urinary 8-hydroxydeoxyguanosine (8-OHdG) levels in elite basketball players.

Petrovic et al. [91] investigated the impact of a four-week chokeberry juice supplementation (100 mL per day) on handball players. They found minor adjustments in the lipid profile and a decrease in TBARS levels in the blood, but these effects were exclusively noted in male participants. Stankiewicz et al. [7] carried out a seven-week study on the effects of consuming 200 mL of chokeberry juice daily (100 mL each in the morning and evening) among young male soccer players. The antioxidant potency of the chokeberry juice was noted to be low, with concentrations of 8.83 mg/mL and 7.62 mg/mL, depending on the method used. They found that this supplementation had no significant impact on the damage caused by free radicals, as indicated by the measurements of TBARS and 8-OHdG levels. Cikiriz et al. [92] conducted a 12-week study on the effects of chokeberry extract supplementation (30 mL per day) on group of handball players. Participants underwent maximal physical exercise on a treadmill before starting the supplementation and received supplementation again after 6 and 12 weeks. The study reported positive changes, including a decrease in TBARS levels and increases in hemoglobin levels, erythrocyte counts, and high-density lipoprotein (HDL) levels, after 6 weeks of supplementation. However, the study did not provide details on the supplement’s composition or its antioxidant capacity. Morillas-Ruiz et al. [93] demonstrated that young cyclists participating in a regulated exercise training regimen experienced enhancements in plasma oxidative stress biomarkers after drinking a beverage rich in polyphenol antioxidants. The beverage included fruit concentrates from black grapes, raspberries, and red currants, with an anthocyanin concentration of 758.6 mg/L. The concentration of TBARS rose immediately following physical exercise and then diminished 45 min after the test. However, attributing these changes solely to polyphenols is challenging because the dietary supplement was composed of berry concentrates rich in polyphenols, carbohydrates, and vitamin C. Additionally, a reduction in TBARS was observed in male rowers who consumed chokeberry juice (containing 23 mg of anthocyanins per 50 mL of juice, taken three times daily for four weeks), which had an anthocyanin content of 23 mg/100 mL [94]. McLeay et al. [95] explored the antioxidant properties of New Zealand blueberries on exercise-induced muscle damage through strenuous eccentric exercises in ten healthy women. The study revealed that consuming a blueberry smoothie with 96.6 mg of anthocyanins before and after exercise-induced muscle damage hastened the recovery of muscle peak isometric strength. This improvement could be attributed to a reduction in the potential for ROS (reactive oxygen species) generation and a gradual enhancement of the plasma’s antioxidant capacity. Park et al. [96] demonstrated that runners experienced an elevation in total antioxidant status (TAS) and a notable decrease in the levels of IL-6 and C-reactive protein (CRP) following physical exercise during a period of blueberry supplementation. Additionally, VO2 max and the duration of exercise performance improved throughout the supplementation phase. The study concluded that blueberry supplementation has the potential to enhance exercise performance and metabolic markers while reducing IL-6 and CRP levels, which are attributed to the increase in TAS levels. Comparable outcomes have been observed in animal research, where blueberry supplementation has shown the ability to enhance physical performance, as evidenced by extended swimming durations, and to reduce oxidative stress [97].

Toscano et al. [98] demonstrated that in recreationally active runners (*n* = 28), consuming the same purple grape juice and dosage for 28 days led to a 38% increase in total antioxidant capacity (TAC) in comparison to a control group. Furthermore, observations on malondialdehyde (MDA) levels indicated that while grape juice supplementation didn’t prevent lipid peroxidation in athletes, the increase was less significant than in participants who didn’t consume grape juice. The researchers deduced that supplementing with purple grape juice can enhance the performance of recreational runners by prolonging endurance, boosting antioxidant activity, and potentially reducing inflammation markers. Tavares-Toscano et al. [99] found that a single serving of purple grape juice (10 mL/kg with a concentration of 3106.6 mg/L) could enhance plasma antioxidant activity in recreational male runners, though it did not affect MDA-induced lipid peroxidation levels. This single dose was shown to have an ergogenic effect by extending the duration runners could maintain exhaustion and increasing antioxidant activity. Another study by Skarpańska-Stejnborn et al. [100] examined the impact of grape polyphenol supplementation on blood antioxidant status by administering three capsules of red grape skin extract daily for six weeks to fourteen physical education students (*n* = 14), with each capsule containing 188 mg/g of polyphenols and 35 mg/g of anthocyanins (malvidin, peonidin, petunidin, delphinidin, cyanidin). The results showed an insignificant modification of antioxidant enzyme activities of SOD, CAT, GSH, and glutathione reductase (GR), concentrations of non-enzymatic antioxidants GSH and uric acid (UA), and total antioxidant status (TAS). However, the authors indicated that the supplementation with the alcohol-free red wine grape polyphenolic extract might influence the attenuation of the post-exercise release creatine kinase (CK) into the blood.

To sum up, the differences in the presented results of studies on the influence of anthocyanin-rich berries on the level of oxidative stress markers after exercise may result from different approaches to the processing and storage of food materials rich in anthocyanins, such as blueberries, blackberries, chokeberries, black raspberries, and purple grape, which may lead to significant anthocyanin losses in the tested food products. Thus, discrepancies in antioxidant efficacy observed across studies may reflect differences in anthocyanin type, food matrix, processing methods, and study design. Future research should aim to standardize intervention protocols and analytical markers. To synthesize and compare the key findings across different anthocyanin sources, populations, and study designs, selected human intervention studies are summarized in Table 2. The table highlights differences in outcomes based on dose, duration, and type of intervention.

## 6. Dietary Recommendations and Research Perspectives

### 6.1. Anthocyanin Content, Structure, and Dietary Exposure

The anthocyanin-rich fruit consumption may be able to reduce exercise-induced oxidative stress [101]. Among purple fruits and vegetables, the highest anthocyanin content is found in red cabbage, black carrots, purple potatoes, and blueberries [6,64]. These compounds are known for their potent antioxidant properties, largely due to a positively charged oxygen atom in their structure. Unmethylated anthocyanins, particularly those with numerous OH groups in the B ring, exhibit greater antioxidant activity compared to their methylated counterparts.

Absorption, metabolism, tissue distribution, and excretion of anthocyanins depend on their chemical structure and food matrix [102,103,104,105,106]. The black currant anthocyanins (delphinidin glycosides) appear to be a more effective antioxidant than blueberry anthocyanins (malvidin glycosides) [102]. The body absorbs a small portion of orally consumed anthocyanins as intact glycosides, which are quickly metabolized by various tissues and organs within 2 h of ingestion [103]. Foods rich in delphinidin, cyanidin, and malvidin anthocyanins are considered promising for their potential health benefits [104]. A different anthocyanin profile can be found in fresh purple fruits and vegetables compared to their processed forms. The quality of anthocyanin content in juices, jams, or other preserves depends on storage, purification, and processing [6].

### 6.2. Intake Recommendations, Efficacy, and Applications in Exercise Nutrition

The European Food Safety Authority (EFSA) notes that the average dietary exposure to anthocyanins is quite low [81]. Estimated daily anthocyanin intake ranges from 3 to 215 mg/day, depending on dietary habits and regional differences [102]. The actual anthocyanin content in samples as a juice, concentrate, natural colorant, and dietary supplements may be lower than the content advertised on the labels [6]. Such discrepancies may mislead consumers and pose potential health risks, particularly if products contain undeclared or inaccurately labeled ingredients.

No adverse effects of anthocyanin consumption have been reported in either humans or animals [103,105]. In China, the recommended daily intake of anthocyanins is 50 mg [103]. Studies have shown that anthocyanin supplementation in the range of 300 to 320 mg per day can reduce pro-inflammatory markers and support systemic anti-inflammatory responses in healthy individuals [87]. Combining anthocyanins with other antioxidants, like vitamin C, can enhance their efficacy. A diet rich in both has been shown to improve antioxidant defenses more effectively than either nutrient alone [107]. Notably, cyanidin-3-glucoside, even at relatively low concentrations (0.1 and 0.01 μmol/L), is capable of inducing eNOS phosphorylation. These concentrations are within the absorption capacity of the human body, making the effect physiologically relevant [87].

The available literature does not provide clear data on what amount (mg) of the total daily dose of anthocyanins from purple food matrix has the greatest impact on reducing oxidative stress and inflammation induced by physical exercise in trained and untrained people. Higher dosages appear to produce better results. To be effective, athletes should consume anthocyanin-rich supplements 2–3 times daily, each dose containing more than 100 mg of anthocyanins [101]. Athletes should consider consuming anthocyanin-rich foods immediately after exercise to reduce muscle soreness and accelerate recovery. Pre-exercise consumption may also offer protective effects against oxidative damage [95]. Antioxidant supplements derived from anthocyanin-rich fruits reduce oxidative stress in anaerobic interventions but not for aerobic interventions [101].

### 6.3. Delivery Innovations and Translational Challenges

Recent advances in food technology and delivery systems have aimed to enhance the bioavailability of anthocyanins, which are otherwise rapidly degraded in the gastrointestinal tract. Techniques such as microencapsulation using biopolymers (e.g., maltodextrin, chitosan, gum arabic) and nanoformulations (e.g., liposomes, nanoemulsions) have shown promise in protecting anthocyanins from pH-induced degradation and enzymatic breakdown [108,109]. Studies have demonstrated that encapsulated anthocyanins exhibit prolonged release, higher intestinal absorption, and improved stability during storage [110]. Co-formulation with phospholipids or probiotics is also under investigation as a strategy to enhance anthocyanin uptake and modulate microbiota-mediated metabolism [111]. These innovations represent a promising direction for the development of functional foods and nutraceuticals with more reliable health effects.

**Figure 4:**
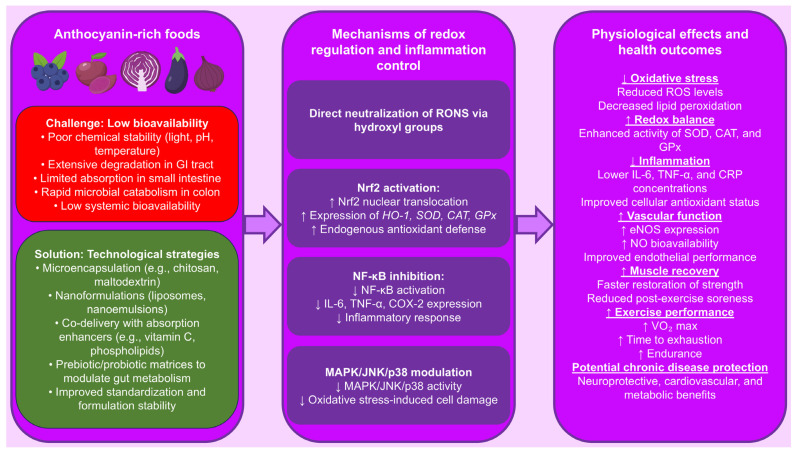
Proposed molecular mechanisms by which anthocyanins modulate oxidative stress and inflammation. The figure integrates three key levels: (1) dietary sources and challenges related to anthocyanin bioavailability, (2) molecular mechanisms underlying antioxidant and anti-inflammatory activity, and (3) systemic physiological effects. Technological strategies to overcome low bioavailability include microencapsulation, nanoformulation, co-delivery with enhancers, and probiotic/prebiotic matrices. Biological effects include direct neutralization of reactive oxygen and nitrogen species (RONS), activation of nuclear factor erythroid 2–related factor 2 (Nrf2), inhibition of nuclear factor kappa B (NF-κB), and modulation of the mitogen-activated protein kinase (MAPK), c-Jun N-terminal kinase (JNK), and p38 signaling pathways. These actions result in enhanced expression of endogenous antioxidant enzymes—superoxide dismutase (SOD), catalase (CAT), glutathione peroxidase (GPx), and heme oxygenase-1 (HO-1)—as well as improved endothelial nitric oxide synthase (eNOS) activity, increased nitric oxide (NO) production, reduced levels of pro-inflammatory cytokines (interleukin 6 [IL-6], tumor necrosis factor alpha [TNF-α] and C-reactive protein [CRP]), improved redox balance, exercise performance, and recovery, and potential protection against chronic diseases, ↑—increase; ↓—decrease.

However, despite these technological advances, several challenges remain. For example, the scalability of encapsulation methods, the cost of production, and the interaction of anthocyanins with food matrices during industrial processing may limit their widespread application. Moreover, comparative studies evaluating the effectiveness of various delivery systems in vivo are still limited. Novel food processing approaches such as fermentation, spray-drying, or high-pressure treatment may also influence anthocyanin stability and bioactivity and warrant further investigation [112]. Figure 4 illustrates the key challenges in anthocyanin delivery, the molecular mechanisms involved in redox regulation and inflammation control, and the resulting physiological benefits.

While the clinical efficacy of anthocyanins is promising, the lack of standardized dosing and variability in product quality remain key challenges for effective implementation in sports nutrition and public health strategies. Table 3 summarizes the approximate anthocyanin concentrations in selected purple plant foods, illustrating the variability across sources such as chokeberries, blueberries, purple carrots, and sweet potatoes.

## 7. Conclusions

This review highlights the profound health effects of anthocyanin-rich purple vegetables and fruits, particularly in enhancing physical performance and reducing exercise-induced oxidative stress. These compounds not only neutralize free radicals but also offer anti-inflammatory benefits, making them vital for exercise recovery and overall cardiovascular health. Regular consumption of anthocyanin-rich foods, such as purple sweet potatoes, chokeberries, blueberries, and purple grapes, has demonstrated significant potential in improving antioxidant capacity and mitigating exercise-induced oxidative damage.

Despite their health benefits, the bioavailability and stability of anthocyanins pose challenges, necessitating further research to optimize their effectiveness in food products and dietary supplements. Understanding the molecular mechanisms through which anthocyanins exert their protective effects, particularly in the context of exercise-induced oxidative stress, will provide deeper insights into their role in promoting health.

Future research should focus on determining the optimal dosage and consumption patterns of anthocyanin-rich foods to maximize their health benefits. Additionally, exploring strategies to enhance the bioavailability of anthocyanins and improve their stability in various food matrices will be crucial in developing more effective functional foods and supplements. Long-term studies are also needed to assess the sustained health effects of regular anthocyanin consumption, including potential risks and benefits.

Comparative studies on different sources of anthocyanins are essential to identify which fruits and vegetables offer the highest health benefits. This information will be invaluable in tailoring dietary recommendations to individual needs, ensuring that consumers can fully leverage the therapeutic potential of these compounds.

In conclusion, incorporating purple vegetables and fruits into the diet offers substantial physiological advantages, particularly for individuals engaged in regular physical activity. These foods play a critical role in reducing oxidative stress, enhancing recovery, and improving overall performance, making them indispensable components of a health-conscious diet. Addressing the current knowledge gaps will enable us to harness the full therapeutic potential of anthocyanin-rich foods, leading to evidence-based dietary strategies that support health, performance, and longevity.

Future studies should also prioritize investigating the long-term physiological effects of anthocyanins in real-world dietary patterns, including their interaction with other dietary components and bioactives. In particular, more randomized controlled trials are needed to determine the comparative effectiveness of different anthocyanin formulations and food matrices under standardized conditions. Additionally, research exploring the role of gut microbiota in modulating anthocyanin metabolism and bioactivity could provide novel insights into individual variability in response. Clarifying these aspects will help develop targeted dietary strategies and functional products that are both efficacious and personalized.

## Figures and Tables

**Table 1:** Main anthocyanins in fruit and vegetables.

Source			Main Anthocyanins	References
Family	Botanical Name	Common Name		
*Brassicaceae*	*Brassica oleracea*, variety *italica*	Purple sprouting broccoli, broccoli sprouts	cyanidin-3-*O*-diglucoside-5-*O-*glucoside in acylated forms: [cyanidin-3-*O*-(*p*-coumaroyl)(sinapoyl)diglucoside-5-*O*-glucoside, cyanidin-3-*O-*(sinapoyl)(feruloyl)diglucoside-5-*O-*glucoside, cyanidin-3-*O-*(sinapoyl)(sinapoyl)diglucoside-5-*O-*glucoside]	[65,66]
	*Brassica oleracea* L. var. *acephala* DC.	Ornamental purple kale	cyanidin-3-(sinapoyl)(feruloyl)-diglucoside-5-glucoside	[67]
	*Brassica juncea*	Purple mustards	cyanidin-3-*O*-diglucoside-5-*O*-glucoside in acylated forms: [cyanidin-3-*O*-(feruloyl)(sinapoyl)sophoroside-5-*O*-(malonyl)glucoside, cyanidin-3-*O*-(diferuloyl)sophoroside-5-*O*-(malonyl)glucoside]	[54]
	*Brassica oleracea* var. *botrytis*	Cauliflower, purple cape, purple of Sicily cauliflower	cyanidin-3-*O*-galactoside cyanidin-3-*O*-glucoside pelargonidin-3-*O*-glucoside delphinidin-3-*O*-glucoside petunidin-3-*O*-glucoside	[68]
	*Brassica oleracea* L. var. *capitata*	Red cabbage (with purplish colored leaves)	cyanidin-3-*O*-diglucoside-5-*O-*glucoside in acylated forms: [cyanidin-3-*O*-(*p*-coumaroyl)(sinapoyl) triglucoside-5-*O*-glucoside, cyanidin-3-*O*-(feruloyl)(sinapoyl)triglucoside-5-*O*-glucoside, cyanidin-3-*O*-(sinapoyl)(sinapoyl) diglucoside-5-*O*-glucoside]	[69]
	*Brassica oleracea*, variety *gongylodes*	Purple kohlrabi	cyanidin-3-*O*-(feruloyl)-diglucoside-5-*O*-glucoside cyanidin-3-*O*-(feruloyl)(sinapoyl) diglucoside-5-*O*-glucoside cyanidin-3-*O*-(sinapoyl)(sinapoyl) diglucoside-5-*O*-glucoside	[46,70]
*Solanaceae*	*Solanum melongena* L.	Eggplants, aubergine	delphinidin-3-*O*-(coumaroyl)rutinoside-5-*O*-glucoside (nasunin) delphinidin-3-*O*-rutinoside-5-*O*-galactoside delphinidin-3-*O*-glucoside delphinidin-3-*O*-rutinoside	[47,71,72]
	*Capsicum annuum*	Purple pepper (sample varieties: purple bell pepper, jalapeno pepper, purple reaper)	delphinidin-3-*O*-glucoside delphinidin-3-*O*-rutinoside delphinidin-3*-O*-(coumaroyl)rutinoside-5-*O*-glucoside delphinidin-3-*O*-(coumaroyl)ruti–noside-5-*O*-glucoside	[73,74]
	*Solanum tuberosum* L. *Solanum tuberosum* L. var. Purple Majesty	Purple flesh potato	cyanidin-3-*O*-rutinoside petunidin-3-*O*-(coumaryl)rutinoside-5-*O*-glucoside peonidine-3-*O*-(coumaroyl)rutinoside-5-*O*-glucoside peonidin-3-*O*-(caffeoyl-*p*-hydroxybenzoyl)sophoroside-5-*O*-glucoside	[4,5,75]
	*Solanum scabrum*	Garden huckleberry	petunidin-3-*O*-(coumaryl)rutinoside-5-*O*-glucoside	[5]
	*Solanum lycopersicum* L. cv. Del/Ros1	Transgenic purple tomato	petunidin-3-*O*-(coumaryl) rutinoside-5-*O*-glucoside malvidin-3-*O*-(coumaryl)rutinoside-5-*O*-glucoside peonidin-3-*O*-(coumaryl)rutinoside-5-*O*-glucoside	[5]
	*Physallis ixocarpa*	Tomatillo, husk tomato, purple tamarillo Tomatillos small purple-tinged fruits	petunidin-3-*O*-(coumaryl)rutinoside-5-*O*-glucoside	[5]
*Convolvulaceae*	*Ipomoea batatas* L.	Purple sweet potato	cyanidin-3-*O*-(caffeoyl)sophoroside-5-*O*-glucoside peonidin-3-*O*-(caffeoyl)sophoroside-5-*O*-glucoside	[76]
*Apiaceae*	*Daucus carota* spp. *sativus* var. atrorubens	Purple carrot	cyanidin-3-*O*-(xylosyl)(feruloyl)(glucosyl)-galactoside, cyanidin-3-*O*-(xylosyl)(glucosyl)galactoside	[48,58]
*Alliaceae*	*Allium cepa*	Red purplette onion	cyanidin-3-*O*-(malonoyl)glucoside-5-*O*-glucoside cyanidin-3-*O*-(acetoyl)glucoside cyanidin-3-*O*-(malonoyl)(acetoyl)glucoside	[72]
*Fabaceae*	*Phaseolus vulgaris*	Purple beans	malvidin-3,5-*O*-diglucoside	[52]
*Poaceae*	*Zea mays* L. var. *rugosa*	Purple corn, sweet corn	cyanidin-3-*O*-glucoside	[55]
	*Triticum aestivum* L.	Purple wheat	cyanidin-3-*O*-glucoside	[71]
*Rosaceae*	*Rubus idaeus* L. *Amethyst (*purple)	Purple and purple–black raspberries	cyanidin-3-*O*-glucoside cyanidin-3-*O*-rutinoside cyanidin-3-*O*-sophoroside cyanidin-3-*O*-(coumaroyl)glucoside	[6,64]
	*Rubus fruticosus* L.	Blackberries	cyanidin-3-*O*-glucoside cyanidin-3-*O*-rutinoside cyanidin-3-*O*-(dioxaloyl)glucoside	[6,77]
	*Prunus domestica* L.	Plum	cyanidin-3-*O*-glucoside, cyanidin-3-*O*-rutinoside, peonidin-3-*O*-glucoside, peonidin-3-*O*-rutinoside	[6,78]
	*Aronia prunifolia* L.	Purple chokeberry	cyanidin-3-*O*-galactoside, cyanidin-3-*O*-glucoside, cyanidin-3-*O*-arabinoside, cyanidin-3-*O*-xyloside	[79]
*Ericaceae*	*Vaccinium myrtillus* L.	Bilberries	cyanidin-3-*O*-glucoside cyanidin-3-*O*-galactoside cyanidin-3-*O*-arabinoside delphinidin-3-*O*-galactoside delphinidin-3-*O*-glucoside delphinidin-3-*O*-arabinoside malvidin-3-*O*-galactoside malvidin-3-*O*-glucoside malvidin-3-*O*-arabinoside petunidin-3-*O*-galactoside petunidin-3-*O*-glucoside petunidin-3-*O*-arabinoside	[6,59,64,71]
	*Vaccinium corymbosum* L.	Blueberry (highbush and low bush)	cyanidin-3-*O*-galactoside, cyanidin-3-*O*-glucoside, malvidin-3-*O*-glucoside, malvidin-3-*O*-galactoside, delphinidin-3-*O*-galactoside, delphinidin-3-*O*-glucoside, petunidin-3-*O*-galactoside, petunidin-3-*O*-glucoside, peonidin-3-*O*-galactoside, peonidin-3-*O*-glucoside,	[6,64]
	*Empetrum nigrum*	Crowberry (black)	cyanidin-3-*O*-glucoside, cyanidin-3-*O*-arabinoside delphinidin-3-*O*-glucoside delphinidin-3-*O*-arabinoside malvidin-3-*O*-glucoside, malvidin-3-*O*-arabinoside peonidin-3-*O*-glucoside, peonidin-3-*O*-arabinoside petunidin-3-*O*-glucoside, petunidin-3-*O*-arabinoside	[6]
*Viburnaecea*	*Sambucus* spp.	Elderberries	cyanidin-3-*O*-glucoside, cyanidin-3-*O*-sambubioside, cyanidin-3-*O*-sambubioside-5-*O*-glucoside	[61,71,77]
*Vitaceae*	*Vitis vinifer* L. Concord	Purple grapes	cyanidin-3-*O*-glucoside, delphinidin-3-*O*-glucoside, malvidin-3-*O*-glucoside, malvidin-3-*O*-(acetyl)glucoside, malvidin-3-*O*-(coumaroyl)glucoside, peonidin-3-*O*-glucoside, petunidin-3-*O*-glucoside	[6]
*Caprifoliaceae*	*Lonicera caerulea*	Honeyberries (haskaps, honeysuckle)	cyanidin-3-*O*-glucoside cyanidin-3-*O*-rutinoside, peonidin-3-*O*-glucoside, cyanidin-3,5-*O*-diglucoside	[77]
*Grossulariaceae*	*Ribes nigrum* L.	Black currant	cyanidin-3-*O*-glucoside cyanidin-3-*O*-rutinoside delphinidin-3-*O*-glucoside delphinidin-3-*O*-rutinoside	[6,64]
*Asteraceae*	*Cynara scolymus*	Artichoke	Cyanidin-3-*O*-(malonyl)glucoside	[80]

**Table 2:** Summary of human intervention studies using anthocyanin-rich foods in exercise-related oxidative stress and recovery.

Population	Anthocyanin Source	Dose and Duration	Outcome Measures	Key Findings	References
Elite basketball players	Purple sweet potato leaves	200 g/day, 2 weeks	8-OHdG, LDL oxidation	↑ Antioxidant status, ↓ DNA oxidation	[90]
Male soccer players	Chokeberry juice	200 mL/day, 7 weeks	TBARS, 8-OHdG	No significant effect	[7]
Handball players	Chokeberry extract	30 mL/day, 12 weeks	TBARS, HDL, RBC	↓ Lipid peroxidation, ↑ HDL, RBC count	[92]
Healthy women	Blueberry smoothie	96.6 mg pre/post-exercise	Muscle strength, TAS	↑ Recovery, ↑ Antioxidant capacity	[95]
Trained runners	Blueberry-based beverage with 15% aronia	140 g fruit pulp per day, for 4 weeks	IL-6, CRP, VO_2_ max	↓ Inflammation, ↑ Performance	[96]
Recreational runners	Purple grape juice	300 mL/day, 28 days	TAC, MDA	↑ Antioxidant capacity, no differences in lipid peroxidation	[98]
Recreational runners	Purple grape juice	Single dose (10 mL/kg)	Plasma antioxidant activity, MDA	↑ Antioxidant activity, no effect on MDA	[99]
PE students	Red grape extract	3 capsules/day, 6 weeks	SOD, CAT, TAS, CK	No change in enzymes, possible effect on CK	[100]

Abbreviations: 8-OHdG—8-hydroxydeoxyguanosine; LDL—low-density lipoprotein; TBARS—thiobarbituric acid reactive substances; HDL—high-density lipoprotein; RBC—red blood cells; TAS—total antioxidant status; IL-6—interleukin 6; CRP—C-reactive protein; VO_2_ max—maximal oxygen uptake; MDA—malondialdehyde; SOD—superoxide dismutase; CAT—catalase; CK—creatine kinase; and TAC—total antioxidant capacity, ↑—increase; ↓—decrease.

**Table 3:** Approximate anthocyanin content in selected purple plant foods.

Plant Source	Approx. Anthocyanin Content (mg/100 g Fresh Weight)	References
Purple sweet potato	286.6	[88]
Chokeberry	500–1000	[94,113]
Blueberry	82.5–530	[95,114]
Grape	8–750	[99,114]
Red cabbage	25–90.5	[114,115]
Purple carrot	0.5–191	[116]
Blackcurrant	130–400	[6,114]
Eggplant	750	[114]

## Data Availability

No new data were created or analyzed in this study. Data sharing is not applicable to this article.
